# VRAM Versus ALT Flap Reconstruction for Large Head and Neck Defects: Does Weight Influence Complication Rate?

**DOI:** 10.3390/jpm14070720

**Published:** 2024-07-03

**Authors:** Courtney B. Shires, Jataiveus Jackson, Jessica Moskovitz, Karuna Dewan

**Affiliations:** 1 West Cancer Center, 7945 Wolf River Blvd, Germantown, TN 71115, USA; 2 Department of Otolaryngology-Head and Neck Surgery, Louisiana State University Health Shreveport, Shreveport, LA 71118, USA; 3 Department of Otolaryngology, Oschner University, Gretna, LA 70112, USA

**Keywords:** ALT, anterolateral thigh, VRAM, vertical rectus abdominus muscle, free flap, obese, body weight

## Abstract

**Background:** Obesity remains a high-level risk factor for various cancers. Yet, some research has shown that higher BMIs may improve survival outcomes, particularly for head and neck squamous cell carcinoma (HNSCC). While this is a clear deviation from the norm, it raises the question of what other effects obesity may have on head and neck cancer patients, including surgical outcomes. Choosing the most appropriate flap for patients can be difficult for even experienced surgeons. Head and neck reconstructive surgeries are no exception to this rule and can be especially challenging. To produce the most favorable outcomes, a physician must be able to combine all flap attributes including donor and recipient site location, flap thickness, as well as each patient’s individual risk factors such as prior radiation. **Purpose:** The purpose of this study was to compare the outcomes of vertical rectus femoris myocutaneous (VRAM) and anterolateral thigh (ALT) flaps in overweight and obese individuals with varying head and neck cancers leaving large-sized defects to the outcomes in normal and underweight patients. **Methods:** A retrospective chart review was conducted of VRAM and ALT flaps performed over a period of 12 months at our university hospital for large head and neck reconstruction. **Results:** Of the 17 ALT patients, 80% (4/5) of the underweight patients, 57% (4/7) of the normal weight patients, 50% (1/2) of the overweight patients, and 33% (1/3) of the obese patients experienced complications. Of the 15 VRAM patients, 40% (2/5) of the underweight patients, 83% (5/6) of the overweight patients, and 50% (1/2) of the obese patients experienced complications. **Conclusions:** In our small sample size, a smaller percentage of obese patients with head and neck cancer who underwent flap reconstruction surgery had complications when undergoing ALT flaps than VRAM flaps, which contrasts with the normal and underweight patients, who had less complications with VRAM flaps than ALT flaps.

## 1. Background

Obesity has long been an epidemic in America [1,2]. While there are various causes and factors that can contribute to obesity, it has been established that obesity increases a patient’s risk for various comorbidities including cancer. For example, there seems to be a near two-fold increase in risk of postoperative complications and/or flap failure in patients with a higher BMI who need breast reconstruction [3]. Although there is a rule of thumb that obesity further promotes negative outcomes in cancer patients, there is growing evidence that obesity improves outcomes in patients with certain types of head and neck cancers (HNCs). Hence, obesity at the time of diagnosis of head and neck squamous cell carcinoma (HNSCC) is associated with increased survival outcomes as well as decreased rates of recurrence [4]. 

Patients with various forms of cancer often require surgical reconstruction using free flaps to repair deficits caused by the disease. When choosing the right flap, a physician must be educated on the common risk factors for flap rejection, while also considering the risks which are specific to certain reconstruction sites or donor areas. For instance, if a patient with oral cancer requires reconstruction, the surgeon would need to consider the effects of the saliva in the oral cavity following the operation. The effects of the saliva can lead to fistulas, infection, and even total flap failure, creating another obstacle that must be considered when choosing the most appropriate flap [5]. Because it has been generally established that obesity confers better outcomes in patients with HNCs, some may now question the way obesity affects this population’s response to specific surgical reconstruction techniques.

Providing a flap that has the best functional outcome while also retaining as much of the natural cosmetics of the area can be a daunting task. Patients who suffer from obesity or tobacco abuse, patients who have had prior radiation therapy, or patients with an intraoral communication defect are all at increased risk of morbidities associated with flap reconstruction surgeries. This is of particular importance for this study because there does not seem to be a substantial amount of research discussing the effects of specific flap types in obese patients with HNCs. Because flap thickness, donor sites, and reconstruction locations are all critical factors for promoting successful reconstruction outcomes and preventing flap rejection, the risks associated with certain flap types in this patient demographic must be carefully considered. 

With the initiation of microvascular anastomosis and free-tissue transfer, the vertical rectus abdominis flap (VRAM) has become possibly the most versatile flap used to reconstruct a wide range of defects from the head and neck, breast, and chest wall to the groin and perineal region. Along with the latissimus dorsi and radial forearm flap, the VRAM flap has become one of the workhorse flaps of reconstructive surgery. A reliable blood supply, the ability to harvest a large size, and a good cosmetic outcome make it a preferred selection by reconstructive surgeons.

The VRAM flap is especially useful in preventing wound-related complications in radiated cancer patients since it has a robust blood supply. This flap can be harvested as a muscle-only flap or as a myocutaneous flap. Contouring the flap by excising the subcutaneous tissue and placing a skin graft over the muscle or debulking the muscle can be useful in the head and neck. A long vascular pedicle can be harvested for anastomosis in the head and neck [6].

In addition to the VRAM flap, the ALT flap has become a very popular free flap for reconstruction of defects of the head and neck. The ALT flap has become a significant option for the simultaneous reconstruction of multiple anatomical sites in head and neck [7]. This flap can be harvested with a very large bulk to include skin only, both skin and muscle, or even as a chimeric flap with separately perfused skin paddles for multiple anatomical locations with a minimal donor site morbidity. These features make the ALT flap ideal for combined defects of the head and neck. Complex defects simultaneously involving the pharynx, cervical esophagus, cervical trachea, tracheal stoma, and anterior neck skin can be safely and reliably accomplished with an ALT flap [8]. Musculocutaneous ALT flaps are useful when bulk is needed to fill large spaces, for instance, in total or near total glossectomy defects. On the other hand, thinning an ALT can be useful in pharyngo-laryngeal reconstruction where excessive bulk of the flap may occlude the tracheostoma [9].

The ALT flap has a few inherent disadvantages. The learning curve can be longer than other commonly used flaps, mostly because the intramuscular perforator dissection is more challenging [10]. While this flap is very versatile and can be used in a variety of defects, the surgeon must become familiar with the anatomical variations. Tubing the skin paddle may remain difficult even after thinning the ALT flap in larger patients [9].

This study aims to investigate the relationship between obesity and the specific free flap types in patients with HNCs and their surgical outcomes. This study also hopes to elucidate benefits and risks associated with using VRAM or ALT flaps in this patient population to better improve level of care, decrease postoperative complications, and improve patient outcomes. 

## 2. Methods

A retrospective review was conducted of all ALT and VRAM flap reconstruction surgeries performed for head and neck defects over a one-year period by the same otolaryngologist and free-flap surgeon at our university hospital. All information was obtained from our hospital medical record system. The study was approved by the Methodist University Hospital institutional review board. 

Patients with mucosal (oral cavity, pharynx, larynx) and non-mucosal (orbital, skin, soft tissue of neck) defects were included. All the patients had stage III or IV head and neck cancer. Only patients with head and neck defects needing reconstructive flaps >90 cm^2^ were included. The ALT and VRAM flaps were chosen for our patient population because these were large defects, and a benefit of these two flaps would be their bulk. Patients who had undergone previous abdominal surgery, such as gastrostomy tube, colostomy, hysterectomy, and exploratory laparotomy, underwent ALT flaps instead of VRAM flaps. Patients without previous abdominal surgery underwent VRAM flaps. 

Postoperatively, all the patients followed the same pharmacologic protocol. We did not allow any caffeine, chocolate, nicotine, pressors, or diuretics (outside of their usual home medications) for the first week after surgery. We started aspirin, 81 mg daily, on postoperative day 1. The patients continued this for 30 days. The patients received the usual weight-based subcutaneous heparin throughout their hospitalization.

The same postoperative instructions were followed by all our patients. Patients were maintained in a tropicana room (80–83 degrees) until discharge. They remained on bedrest for the first 48 h after surgery. Subsequently, patients were allowed to get out of bed to a chair on postoperative day (POD) 2 and ambulate on POD 3. They underwent hourly flap checks until 48 h postoperatively, then they underwent every 2 h flap checks until 72 h postoperatively. On POD 4, we advanced to every 4 h flap checks, which were maintained until discharge. Trained nursing staff were allowed to perform flap checks. Doppler wires were cut 2 cm from the skin edge just before the patient was discharged from the hospital.

Our patients were not allowed to eat by mouth for 3–6 weeks postoperatively, depending on their surgical defect and their healing status. In patients with tracheostomies, we did not place trach ties around their necks until POD 5. On POD 3, if patients were progressing well, they were transferred to the regular floor from intensive care unit ICU. We started oral care with peridex and sponge sticks on POD3. Tube feeds began on POD2 with the presence of bowel sounds. On POD 3, intravenous fluids were weaned. No sutures were cut before the patients were discharged home. After they were discharged home from the hospital, we generally evaluated the patients in our clinic weekly.

Patients were considered to be “overweight” if they had a BMI between 25 and 29.9 kg/m^2^, and “obese” if they had a BMI > 30 kg/m^2^. Outcome measures included patient age, gender, BMI, past surgeries, prior chemotherapy and/or radiation, comorbidities (including smoking history), cancer diagnosis, and staging. Patients were then monitored for postoperative complications over a 30-day period. These complications were described as “major” or “minor”. Minor complications included cellulitis (requiring antibiotics, not surgery) and hematoma/seroma. Major complications included wound dehiscence (requiring surgical intervention), flap necrosis, abscess (requiring surgical intervention), and death. 

## 3. Results

### 3.1. Demographics

There were 32 patients in total, 20 males and 12 females. A total of 17 patients underwent ALT flap reconstruction, while the other 15 underwent VRAM flap reconstruction during the study (Table 1). Of the 17 ALT patients, 5 patients were overweight (BMI, 25–29.9 kg/m^2^) or obese (BMI, >30 kg/m^2^). Of the 15 VRAM patients, 8 were overweight or obese. The VRAM patients had a median age of 63 years in both the complications and the no complications group. The median overall age was 63 years, and the average age was 60 years (range 49–88 years). The ALT patients had a median age of 62 years in the no complications group, and 60 years in the complication group. The median overall age was 60 years, and the average age was 62 years. Reconstructions were performed for various cancer defect sites such as the mandible/oral region (n = 24), pharynx/larynx (n = 5), and defects with no intraoral communication (n = 5). Some patients had both mandible/oral and pharynx/larynx defects.

The average size of the defects was large (96 cm^2^ in the VRAM group and 92 cm^2^ in the ALT group). Most patients in our group (75%) had their oral cavity/mandible defects repaired. Up to 10 of these patients underwent VRAM repair, while 14 patients underwent ALT repair. In the VRAM repair group, six patients underwent marginal mandibulectomy and three underwent segmental mandibulectomy. In the ALT repair group, six patients underwent marginal mandibulectomy, while two underwent segmental mandibulectomy. The patients who underwent segmental mandibulectomy had their repair with titanium plate and screws, not a free bone graft. 

### 3.2. Complications According to Weight

The overall rate of complication was similar between the VRAM group (53%) and the ALT set of patients (59%) (Table 2). Of the 17 ALT patients, 80% (4/5) of underweight patients, 57% (4/7) of normal weight patients, 50% (1/2) of overweight patients, and 33% (1/3) of obese patients experienced complications. Of the 15 VRAM patients, 40% (2/5) of the underweight patients, 0/2 (0%) of the normal weight patients, 5/6 (83%) of the overweight patients, and 1/2 (50%) of the obese patients experienced complications. 

### 3.3. Types of Complications

In total, two ALT patients experienced minor complications, and eight experienced major complications including one death (Table 3). Of the 15 VRAM patients, 4 VRAM patients had minor complications, and 4 had major complications, including 1 death. 

The median length of surgery in VRAM was similar between those patients with and without complications (complications = 12 h, no complications = 13 h). However, for ALT flaps, patients with complications had longer median surgery times (16 h) than those without complications (10.5 h). In addition, these patients had longer median surgery times than all the patients undergoing VRAM flap. The average postoperative hospital length of stay for ALT flaps did not differ when compared to VRAM flaps; however, patients with VRAM complications did stay longer, on average, than any other group (16.5 days).

### 3.4. Complications According to Primary Defect Location

The four major complications from VRAM flaps were found in one patient with an oral cavity/mandible defect, one patient with a larynx/pharynx defect, and two patients with non-mucosal defects (Table 4). The four minor postoperative complications after VRAM flaps were in one patient with an oral cavity defect, two with larynx/pharynx defects, and one with a non-mucosal defect. The eight major complications after ALT flaps were found in three patients with oral cavity/mandible defects, four patients with larynx/pharynx defects, and one patient with a non-mucosal defect. The minor complications after ALT flaps were in one patient with a larynx/pharynx defect and in one patient with a soft tissue of the cheek and orbit defect.

### 3.5. Comorbidities

Most of the patients in our study had significant comorbidities (Table 5). We included the following as major comorbidities: diabetes, peripheral vascular disease, coronary artery disease, significant tobacco use history, and previous radiation to the head and neck. In our VRAM patients that experienced complications (n = 8), 75% had three or more major comorbidities. Of the ALT patients that experienced complications (n = 10), 80% had three or more major comorbidities. Interestingly, 86% of both the VRAM patients and ALT patients who did not experience major complications had three or more major comorbidities.

## 4. Discussion

Although obesity does improve the survival outcomes in patients with HNCs, it can pose a challenge for surgeons performing reconstructive surgery, and special care must be taken when choosing the appropriate soft tissue type. For years, many surgeons have preferred the use of rectus flaps for most reconstructive surgeries. It has been noted that obese patients often have increased operative times and overall donor site complications (including flap failure and infection) when using these flaps, particularly for breast reconstructive surgery [11]. The recent literature suggests that BMI has no significant effect on the rate of flap failures for head and neck reconstruction [12]. However, some literature claims that the type of flap used does seem to have an effect. Specifically, the use of ALT flaps is superior to rectus flaps in both obese and overweight patients for a multitude of defects [13] and decreases the overall risk of complications in this patient demographic. Providing the best possible patient outcomes is the goal for every physician, and it has become evidently clear that choosing the right flap for each patient is one of the best ways to ensure this goal. 

In our region, our patient population included a large subset of overweight/obese patients. These patients with large defects after head and neck cancer resection also needed large flaps in repair. We chose ALT and VRAM flaps for our population due to their bulk. In our study, weight had an effect on flap success. For instance, the higher the weight class our ALT patients reached, the less likely the patients were to have major or minor complications. The underweight patients had the highest complication rate, and the obese patients had the lowest complication rate. This effect was the opposite in our VRAM patients. For instance, the normal and overweight patients had the lowest rates of complications after VRAM flap, while the overweight and obese patients had the highest rates of postoperative complications.

We had twice as many major complications with ALT flaps as VRAM flaps, but one death with each flap. Each of the two patients that experienced fatality had more than three major comorbidities. The number of patients in our group that experienced complications after either an ALT or VRAM flap was very similar with regards to patient comorbidities. The patients with and without greater than three comorbidities had nearly equal postoperative complications. This is in congruence with the morbidity that head and neck cancer innately carries, regardless of patient characteristics.

Most of our surgeries performed in this series involved the removal of a portion of the mandible and entry into the oral cavity. The major and minor complications did not seem to have any correlation to defects of the mandible. Laryngeal/pharyngeal defects had a higher rate of major complications, especially after introduction of the ALT flaps, that were most likely secondary to a pharyngocutaneous fistula. 

While ALT flaps in our group resulted in a higher rate of major complications, VRAM flaps in our hands resulted in a higher rate of minor complications. These were, most commonly, local wound infections needing antibiotics. 

Several features of the anterolateral thigh (ALT) flap make it very advantageous in head and neck reconstruction. These include great versatility in skin paddle design, exceptional pedicle length, and, most importantly, the ability to harvest varying amounts of skin, subcutaneous tissues, fascia, and muscle [14]. On the other hand, the VRAM flap can be particularly thick in obese patients due to the pattern of central abdominal rather than the peripheral obesity seen in the usual overweight patient [13]. The ALT flap can be safely thinned by maintaining a constant amount of tissues surrounding the perforating vessels [15], while similar thinning and trimming of the VRAM flap can be challenging and associated with increased rates of flap complications [13].

While a majority of the studies regarding free flap reconstruction have demonstrated increased complications with obesity and increasing BMI, a study from MD Anderson did not find any association between BMI and overall, recipient-site, or donor-site complications for head and neck free flap reconstruction. Interestingly, the study showed that BMI affected flap selection, with more obese patients undergoing RFFF and other flaps compared with patients with lower BMI, the majority of whom underwent ALT flap reconstruction. In contrast, patients in our study with a higher BMI more often underwent ALT flap placement than other free flap reconstruction. While the lower BMI patients at MD Anderson commonly underwent ALT flaps, our lower BMI patients more often underwent VRAM reconstruction [16].

Similar to our study, Mirza showed that when using the VRAM flap, increasing BMI was strongly associated with increased odds of flap necrosis, with overweight patients having 2.5 times (OR, 2.5; 95%CI, 0.62 to 10.073), obese patients 1.8 times (OR, 1.8; 95%CI, 0.390 to 8.401), and morbidly obese patients having up to 9.4 times increased odds of flap necrosis [6].

One concern when using a VRAM flap is developing abdominal wall hernias at the donor site, especially in obese patients. This concern has mostly been alleviated by the closure technique. Reported rates of this complication vary between 0 and 11%. Most surgeons use a prolene mesh to reinforce the abdominal wall, which prevents this complication. McMenamin reported an incisional hernia rate of 19%, but these patients were closed using a nylon suture and no mesh. Mirza used mesh for closure and had no cases of postoperative incisional hernia [6]. Mesh was used in closure of all the VRAM donor sites in our series with no development of abdominal wall hernias, regardless of patient BMI. Therefore, it is recommended to use a mesh reconstruction of the donor site, especially in patients where a large flap is harvested, and a tight closure is expected. 

Significant limitations of this study include the small sample size and the fact that it was a retrospective study. We are interested in using these data to move forward with a prospective study with higher patient numbers in order to obtain statistical significance. We realized that there were more patients that were overweight or obese in the VRAM group compared to the ALT group. However, the patients in the ALT group had a higher BMI average. Also, our patients were not stratified based on commodities, but instead by a history of previous abdominal surgery.

## 5. Conclusions

In our small sample size, a smaller percentage of obese head and neck cancer patients undergoing flap reconstructive surgery had less complications when undergoing ALT flaps versus VRAM flaps. In contrast, a smaller percentage of underweight patients and normal weight patients had complications with VRAM flaps in comparison to overweight and obese patients. Primary defect location or involvement of the mandible did not influence the rate of complications.

## Figures and Tables

**Table 1:** Patient demographics and Results.

	VRAM (n = 15)	ALT (n = 17)
Gender		
M	9	11
F	6	6
Mean age	60 years	64 years
Mean weight		
Obese	3	2
Overweight	5	3
Normal/underweight	7	12
Average defect size	96 cm^2^	92 cm^2^
Defect location		
Oral cavity/mandible	10	14
Marginal mandibulectomy	3	2
Segmental mandibulectomy	6	6
Larynx/pharynx	3	2
Nonmucosal (orbit, skin, soft tissue neck)	2	3

**Table 2:** Complications among weight groups.

	VRAM	ALT
Complications	8/15 (53%)	10/17 (59%)
Obese	1/2 (50%)	1/3 (33%)
Overweight	5/6 (83%)	1/2 (50%)
Normal	0/2 (0%)	4/7 (57%)
Underweight	2/5 (40%)	4/5 (80%)

**Table 3:** Types of complications.

	VRAM	ALT
Major	4	8
Death	1	1
Wound dehiscence needing surgery	2	3
Flap necrosis	0	2
Abscess needing surgery	2	2
Minor	4	2
Cellulitis needing antibiotics	3	1
Hematoma/seroma	1	1

**Table 4:** Complications according to primary defect location.

	VRAM (n = 8)	ALT (n = 10)
Major	4	8
Oral cavity/mandible	1	3
Larynx/pharynx	1	4
Non-mucosal (orbit, skin, soft tissue neck)	2	1
Minor	4	2
Oral cavity/mandible	1	0
Larynx/pharynx	2	1
Nonmucosal (orbit, skin, soft tissue neck)	1	1

**Table 5:** Comorbidities in patients with and without complications.

	VRAM Patients with Comorbidities	ALT Patients with Comorbidities
Patients with complications	6/8 (75%)	8/10 (80%)
Patients without complications	6/7 (86%)	6/7 (86%)

## Data Availability

The data that support the findings of this study are openly available upon request from the authors.

## Abbreviations

Head and neck squamous cell carcinoma (HNSCC); vertical rectus femoris myocutaneous (VRAM); anterolateral thigh (ALT); head and neck cancers (HNC); intensive care unit (ICU); postoperative day (POD).
